# Allergies and major depression: a longitudinal community study

**DOI:** 10.1186/1751-0759-3-3

**Published:** 2009-01-26

**Authors:** Scott B Patten, Jeanne VA Williams, Dina H Lavorato, Michael Eliasziw

**Affiliations:** 1 Department of Community Health Sciences, University of Calgary, Calgary, Canada

## Abstract

**Background:**

Cross-sectional studies have reported associations between allergies and major depression but in the absence of longitudinal data, the implications of this association remain unclear. Our goal was to examine this association from a longitudinal perspective.

**Methods:**

The data source was the Canadian National Population Health Survey (NPHS). This study included a short form version of the Composite International Diagnostic Interview (CIDI-SF) to assess major depression and also included self report items for professionally diagnosed allergies of two types: non-food allergies and food allergies. A longitudinal cohort was followed between 1994 and 2002. Proportional hazards models for grouped time data were used to estimate unadjusted and adjusted hazard ratios.

**Results:**

A slightly increased incidence of non-food allergies in respondents with major depression was observed: adjusted hazard ratio 1.2 (95% 1.0 – 1.5, p = 0.046). Some evidence for an increased incidence of major depression in association with non-food allergies was found in unadjusted analyses, but the association did not persist after multivariate adjustment. Food allergies were not associated with major depression incidence, nor was major depression associated with an increased incidence of food allergies.

**Conclusion:**

Findings from the present study support the idea that major depression is associated with an increased risk of developing non-food allergies. An effect in the opposite direction could not be confirmed. The observed effect may be due to shared genetic factors, epigenetic factors, or immunological changes that occur during depression.

## Background

An association between allergies and depressive disorders has been suspected for many years. However, early studies typically used clinical subjects [1] and/or non-standard psychiatric measurement strategies [2]. Only a few epidemiological studies have examined associations between allergies and psychiatric symptoms in non-clinical populations. A recent cross-sectional study in Canada confirmed that an association exists between self-reported allergies and depressive disorders in the general population [3].

Wamboldt et al. [4] studied depressive symptoms in people with allergies in a mail survey of a Finnish twin sample. The survey included the Beck Depression Inventory [5] and a series of questions about atopic disorders. Subjects reporting three or more atopic disorders were found to have an elevated probability of exceeding a threshold score on the Beck Depression Inventory. Co-twins of subjects reporting three or more atopic disorders also more frequently had elevated depression scores; however, there was no difference between dizygotic and monozygotic twins. Nevertheless, modelling results were interpreted as providing support for shared familial vulnerability. The idea of shared genetic vulnerability received additional support from a study using data on parent-child dyads in the National Health Interview Survey in the US. In this study, an association between parental depressive disorders and atopy in the children was found in biological, but not adoptive, dyads [6].

Timonen et al. [7] studied a 1966 Finnish birth cohort in an investigation that included skin tests to three common allergens as well as several questions about depression and two depression rating scales. Compared to women with negative skin tests, women with positive skin tests were slightly more likely to report that they had been diagnosed with depression by a doctor (6.9% versus 4.3%), but no difference was found in men. However, a subsequent report cast some doubt on whether the association is unique to women. When depressive symptom ratings were incorporated along with physician diagnoses in the definition of depression, a significant association was also observed in men with high symptom levels [8]. Another report from the same study indicated that maternal atopy (assessed indirectly using a questionnaire completed by the proband) was a risk factor for depression in the proband [9].

Another link between allergies and mood was suggested by the observation, based on analysis of a suicide registry, that there is a springtime preponderance of suicides in people previously hospitalized for atopic disorders [10]. Finally, a case-control study investigating the prevalence of cognitive dysfunction in people with allergic rhinitis also reported elevated depression symptom ratings in the allergic rhinitis cases [11].

Various potential explanations for the epidemiologic association between depression and allergies have been put forward. Some of these explanations imply shared etiologic factors (especially shared genetic vulnerability), others an effect of depression on allergy risk and others an effect of allergies on depression risk. Any one, or all, of these alternatives could explain the associations reported in cross-sectional studies. However, they would have different implications for the longitudinal epidemiology. The most prominent explanations have involved cytokine-related mechanisms [10]. For example, shared vulnerability factors may include genes involved in immune regulation, consistent with the epidemiological findings of Wambold [4] and Timonen [9]. On the other hand, it has been asserted that the brain may interpret immune activation much as if it were a stressor [12], thereby triggering a stress response that may include depression. Longitudinally, this would suggest that allergies, which involve immune activation, may alter the risk of major depression. Depressive disorders are associated with elevated levels of pro-inflammatory cytokines such as interleukin-1β, interleukin-6, tumour necrosis factor-α and interferon-α [12]. These signalling molecules may potentially contribute to the aetiology of some types of allergies through their pro-inflammatory effects. Responses to stressors and immune responses may share underlying mechanisms [12], so it is also possible that epigenetic mechanisms (e.g. alteration of chromatin structure through effects on DNA methylation lead to a lasting sensitization of stress response systems following pre-natal or post-natal stress exposure [13]) could increase the risk both of developing major depression and developing allergies. In the case of food allergies, stressors may also contribute to intestinal hypersensitivity by altering transepithelial permeability, see a brief review of possible mechanisms by Buret [14]. However, there is a consensus that most self-reported food allergies in the community are not attributable to Type I or Type IV hypersensitivity [15], leading to the suspicion that there may be false positive reports based on a misinterpretation of somatic symptoms. These could also be related to depressive disorders, since somatic symptoms can be a manifestation of depression and anxiety. Finally, since most chronic conditions are associated with depressive disorders [16], it is possible that allergies may increase depression risk through the stressful psychological effects of the illness experience.

The extent of epidemiological data concerning the depression-allergy association is surprisingly limited. The Finnish studies used self-reported lifetime depression, hospital admission records or symptom rating scales to assess depression. None of these measurement strategies are generally considered adequate for psychiatric epidemiological research. Generally, structured diagnostic interviews are the preferred approach. Depressive symptom ratings tend to be neither sensitive nor specific for clinically significant Axis I disorders such as depressive disorders. The main deficit in the existing literature is the paucity of longitudinal data. As noted above, it is possible that allergies may increase depression risk or that depression may increase allergy risk, two distinct possibilities that cannot be distinguished using cross-sectional data. In order to address some of the weaknesses of prior studies and to explore the etiological issues noted above, we undertook an evaluation of the longitudinal epidemiology of these variables using data collected in a large scale population survey in Canada.

## Methods

### Study Design

The NPHS is based on a nationally representative community sample assembled by Statistics Canada (Canada's national statistical agency) in 1994. Detailed information about the methods employed in this study may be found on the Statistics Canada Web page . In brief, the National Population Health Survey (NPHS) commenced in 1994 as a cross-sectional survey of Canadian household residents, excluding some specific groups (e.g. First Nations peoples living on reserves, armed forces personnel) and some remote areas. The NPHS sample has been re-interviewed every second year in subsequent cycles. The initial interviews were conducted face to face, but subsequent cycles have collected data predominantly by telephone. An attrition analysis for the NPHS was reported by Beaudet et al. [17]. Attrition was found to be related to several variables, but not to major depression. This is consistent with the literature of prospective psychiatric epidemiological studies. Major depression may increase loss to follow-up by mechanisms such as "unable to locate," but tends to decrease the frequency of refusal, such that the net effect on attrition is weak [18,19]. Statistics Canada produces a longitudinal data file that is made available to researchers through Regional Data Centres, including one located on the University of Calgary campus where the current analysis was conducted.

### Participants

The longitudinal cohort included 17, 276 participants, but the current analysis was restricted to n = 15,254 respondents who were over the age of 12 at the time of the initial 1994 interview. The youngest of these respondents would have been approximately 20 years old in 2002 at the end of the follow-up interval examined in this study.

### Measures

The NPHS interview includes a series of items concerned with long-term medical conditions. Study participants were read a list of chronic medical conditions and asked whether they had been diagnosed with one of these conditions by a health professional. The wording of the relevant item was: *Now I would like to ask about certain chronic health conditions which you may have. We are interested in long-term conditions that have lasted, or are expected to last, 6 months or more and that have been diagnosed by a health professional*. One of the subsequent items was, *Do you have food allergies? *This was followed by, *Do you have any other allergies?*

The interview also includes the Composite International Diagnostic Interview short form (CIDI-SF) [20] for major depression, which assesses past year major depressive episodes. In distinction to rating scales, the CIDI-SF is a condensed structured diagnostic instrument. It contains branches, so that the questions asked depend on responses to earlier items. The CIDI-SF uses a point-based scoring algorithm that incorporates the number of symptom-based criteria fulfilled and the necessity for at least one of two key symptoms (depressed mood and loss of interest or pleasure) in keeping with the Diagnostic and Statistical Manual of Mental Disorders (DSM-IV) [21]. A score of 5 on the CIDI-SF is also consistent with DSM-IV-defined MD on the basis of face validity since the manual requires fulfilment of five of nine specified symptoms, including at least one of the two key symptoms. Furthermore, this cut-point maximized performance of the CIDI-SFMD in a DSM-IIIR-based receiver operator curve analysis carried out during the instrument's development, where it was associated with a 90% positive predictive value for major depression [20]. Due to a lack of "organic" and hierarchical exclusion items, the CIDI-SFMD may be vulnerable to false positive ratings. Consistent with this idea, experience with the instrument suggests that it may slightly overestimate prevalence [22]. However, any such effects must be modest in magnitude, as the CIDI-SFMD has produced credible estimates during applications in Canada [23,24], the US [25,26] and elsewhere [27]. In the NPHS and CCHS surveys the CIDI-SMFD has consistently replicated the expected pattern and strength of association with demographic and clinical variables [24,28-30]. Furthermore, incidence estimates from the CIDI-SFMD [29,30] are consistent with those of a systematic review of high quality studies by Waraich et al. [31].

The NPHS also assessed smoking status and inquired into a series of significant life stressors. These items were preceded by the statement: *The next few questions ask about some things that may have happened to you while you were a child or a teenager, before you moved out of the house. Please tell me if any of these things have happened*. This was followed by a list of seven stressful events: spending 2 weeks or more in hospital, parents getting a divorce, prolonged parental unemployment, something "scary" happening that was thought about for years after, being sent away for doing something wrong and parental alcohol or drug abuse. Respondents answering affirmatively to any one of these items were coded as positive for childhood stressors. Because one of these stressors, prolonged hospitalization, could be associated with allergies or depression, the analysis was repeated not-including this particular item. This had no effect on the results and these analyses are not reported.

### Data Analysis

For the current analysis, one objective was to evaluate the incidence of depressive episodes in people with allergies. This was accomplished by excluding those respondents having an episode of major depression at or in the year preceding the initial interview (n = 849). We also excluded those who did not complete the CIDI-SF in 1994 (n = 1,230). We then assessed the frequency of subsequent episodes in n = 13,175 respondents without major depression at the baseline interview. The incidence of allergies was assessed by excluding the respondents who reported allergies at the baseline interview (n = 2,641) or had missing data on this variable (n = 23) and then comparing the frequency of new onset allergies in those with and without depression. Similar analyses were conducted to assess the incidence of allergies in those with a depressive episode at any of the four available survey interviews during follow-up, and to assess the incidence of food allergies in those with major depression and the incidence of depressive episodes in those with food allergies.

We initially calculated two-year incidence for the first period of follow-up (1994 to 1996) and then stratified these estimates by age and sex. Subsequently, proportional hazards models were used to estimate the hazard ratio for the entire 1994 to 2002 follow-up interval with and without adjustment for potential confounding variables. Childhood stressors were considered potential confounders, based on data from New Zealand indicating that these may be risk factors for asthma [32]. Smoking is another variable that may act as a confounder because it is a risk factor for depression and may protect against respiratory allergies [33]. The proportional hazards model was fit as a generalized linear model of the binomial family with a complementary log-log link, as described by Jenkins [34]. Age and sex were also included in these models.

The NPHS used a multistage, stratified cluster design to select eligible households. To correct the potential bias resulting from this complex survey design, Statistics Canada recommends a bootstrap procedure using a set of replicate weights that they supply. All results presented here were produced with this approach and are therefore representative of the targeted population. The standard error associated with specific estimates, p-values and confidence intervals (CIs) are adjusted for survey design effects by the bootstrap weighting procedure. All analyses were conducted using STATA [35]. The study received approval from the University of Calgary Conjoint Ethics Board.

## Results

Table 1 presents crude and stratified two-year incidence for non-food allergies in the first NPHS cycle, between 1994 and 1996. The incidence was very high. The overall incidence was 14.1% during this two year interval (95% CI 13.1 – 15.0), and was slightly higher in those with major depression at baseline, in women, in the younger age categories, in non-smokers and those reporting childhood stressors, see Table 1. The pattern of higher incidence in women and in younger age groups is consistent with prior studies [33].

**Table 1:** Two-year non-food allergy incidence in the NPHS* 1994 to 1996, stratified by potential predictive variables

	Allergy Incidence % (95% CI)
Total Major depression	14.1 (13.1 – 15.0) 19.3 (14.9–23.7)
No major depression	13.8 (12.8–14.8)
Male	11.1 (9.9–12.3)
Female	16.9 (15.4–18.3)
Age 12–18**	16.7 (12.6–20.7)
Age 19–25	16.4 (13.5–19.3)
Age 26–45	14.5 (12.9–16.0)
Age 46–65	13.3 (11.7–14.9)
Age 66+	10.3 (8.3–12.4)
Daily smoker	13.7 (11.8–15.6)
Never/former/occasional smoker	14.3 (13.3–15.4)
Reported childhood or adult stressors	15.9 (14.6–17.3)
No childhood or adult stressors reported	12.0 (10.8–13.2)

* National Population Health Survey

** age at the baseline interview in 1994

While non-food allergy incidence was very high in the first cycle, it declined during each subsequent NPHS cycle, with 2-year incidence being 8.5% (95% CI 7.6 – 9.3) between 1996 and 1998, 6.9% (95% CI 6.0 – 7.7) between 1998 and 2000 and 6.1% (95% CI 5.3 – 6.9) between 2000 and 2002. The crude hazard ratio (HR) for the effect of major depression at baseline (measured in 1994) on allergy incidence was 1.4 (95% CI 1.2 – 1.6, p < 0.001). No significant interaction terms were observed in the proportional hazards models, but significant effects were seen for sex (HR = 1.5, p < 0.001), smoking (HR = 0.8, p = 0.02) and childhood and adult stressors (HR = 1.2, p =< 0.001). After adjustment for all variables in Table 1, including age, the HR for the effect of major depression at baseline on non-food allergy incidence was 1.2 (95% 1.0 – 1.5, p = 0.046), see Table 2.

**Table 2:** Proportional hazard model for non-food allergy incidence, by major depression status

Variable	Coefficient	Hazard Ratio	Z Statistic	P-value	95% CI		
Major Depression	0.22	1.2	1.99	0.046	1.0	-	1.5
Female	0.41	1.5	6.71	<.001	1.3	-	1.7
Age 12–18	0.41	1.5	1.71	0.087	0.9	-	2.4
Age 19–25	0.59	1.8	5.53	<.001	1.5	-	2.2
Age 26–45	0.41	1.5	4.68	<.001	1.3	-	1.8
Age 46–65*	0.31	1.4	3.38	0.001	1.1	-	1.6
Smoking	-0.17	0.8	-2.40	0.017	0.7	-	1.0
Childhood Stressors	0.22	1.2	4.00	<.001	1.1	-	1.4
Intercept	-2.48	-	-	-	-		-

* age > 65 in 1994 is the baseline category

As major depression is often conceptualized as a chronic, recurrent condition [36], it is meaningful to examine the incidence of allergies in respondents having an episode at any time during the NPHS follow-up. The results were similar to the previous analysis. Here, the adjusted HR for the effect of depression on non-food allergy incidence was 1.3 (95% CI 1.1 to 1.6).

In the first NPHS cycle (1994 to 1996) the two-year incidence for major depression was 3.5% (95% CI 3.1 – 4.1). The 2-year incidence was 4.2% among those with non-food allergies (95% CI 3.1 – 5.3) at baseline and 3.4% in those without (95% CI 2.9 – 3.9). The 2-year incidence in relation to demographic and clinical factors followed the expected pattern, see Table 3.

**Table 3:** Two-year incidence of major depression in the NPHS* 1994 to 1996, stratified by potential predictor variables

	Depression Incidence % (95% CI)
Non-food allergies	4.2 (3.1 – 5.3)
No non-food allergies	3.4 (2.9 – 3.9)
Male	2.6 (1.9–3.3)
Female	4.4 (3.8–5.0)
Age 12–18**	5.7 (3.8–7.6)
Age 19–25	3.4 (2.3–4.5)
Age 26–45	4.0 (3.2–4.8)
Age 46–65	2.6 (1.9–3.3)
Age 66+	1.6 (0.9–2.4)
Daily smokers	5.9 (4.6–7.1)
Never/former/occasional smoker	2.6 (2.2–3.1)
Childhood or adult stressors reported	4.6 (3.8–5.4)
No childhood or adult stressors reported	2.0 (1.6–2.5)

* National Population Health Survey

** age at the baseline interview in 1994

Over the entire follow-up interval, an association between non-food allergies at baseline and major depression incidence was also observed, but only in unadjusted analyses. The crude HR was 1.4 (95% CI 1.2 – 1.6, p < 0.001). After adjustment for the variables listed in Table 3 the HR diminished to 1.2 and was no longer statistically significant (95% CI 0.9–1.4, p = 0.16). In these analyses, no interactions were observed, but effects of sex, age and childhood stress remained significant, see Table 4. Smoking had the expected risk-elevating effect on major depression incidence in this model (HR = 1.8, 95% CI 1.5 – 2.1), see Table 4.

**Table 4:** Proportional hazard model for major depression incidence

Variable	Coefficient	Hazard Ratio	Z Statistic	P-value	95% CI		
Non-Food Allergies	0.14	1.2	1.41	0.16	0.9	-	1.4
Female	0.44	1.6	5.14	<.001	1.3	-	1.8
Age 12–18	1.15	3.2	2.82	<.001	1.4	-	7.1
Age 19–25	1.02	2.8	5.17	<.001	1.9	-	4.1
Age 26–45	0.86	2.4	4.80	<.001	1.7	-	3.3
Age 46–65*	0.57	1.8	2.98	<.001	1.2	-	2.6
Smoking	0.57	1.8	6.47	<.001	1.5	-	2.1
Childhood Stressors	0.71	2.0	8.08	<.001	1.7	-	2.4
Intercept	-0.20	-	-	-	-		-

* age > 65 in 1994 is the baseline category

The remaining analyses examined associations involving food allergies rather than non-food allergies. The crude HR for the effect of major depression at baseline on subsequent food allergy risk was 1.4 (95% CI 1.1 – 1.8). After adjustment for the factors listed in Table 1 the HR was diminished to 1.2 (0.9 – 1.5, p = 0.33). Childhood stressors were significantly associated with food allergy risk (HR = 1.4, 95% CI 1.2 – 1.7, p < 0.001). Neither the crude (hazard ratio 1.2, 95% CI 1.0 – 1.5, p = 0.10) nor adjusted hazard ratio (HR = 0.9, 95% CI 0.7 – 1.3, p = 0.75) suggested an effect of food allergies on major depression incidence.

## Discussion

In this analysis, an elevated incidence of non-food allergies was found in respondents with major depression. An effect of smoking on depression incidence was seen, and smoking was found to protect against the incidence of non-food allergies, as reported by Hjern et al. [33]. These authors postulated that a suppressant effect of smoking on immune function, or physical barriers in the lungs caused by smoking-induced mucosal oedema could explain this apparent protective effect [33]. Childhood stressors were also associated with allergy incidence. No effect of depression on the development of food allergies, or effect of food allergies on depression incidence was observed.

Whereas cross-sectional studies cannot distinguish an effect of major depression on allergy risk from an effect of allergies on major depression risk, the longitudinal design employed here provided an opportunity to do so. Evidence of an effect of major depression on allergy risk was found, but the evidence for an effect in the opposite direction could not be confirmed after adjustment for potential confounding variables. As such, the results of this study primarily provide support for the idea that major depression is associated with an increased risk of allergies. This could occur either because depression increases the risk of allergies (perhaps because of immune system changes that occur in depression, see discussion above) or because some other risk factor is a determinant both of major depression and allergies. Existing literature suggests that genetic predisposition may be a determinant of both conditions. Whereas genetic predisposition could not be assessed in this study, another candidate as a shared risk factor, childhood stressors, was measured and the association of major depression with allergy risk persisted after adjustment for this variable. Childhood stressors could trigger epigenetic mechanisms leading to long-term activation of the stress response [13]. In turn, in view of physiological relationships between stress and immune responses [12], epigenetic changes could be a shared determinant of depression and allergies. Although the association between major depression and allergy persisted after adjustment for childhood stressors, direct measures of stress and immune responses were not made, so the results cannot support or refute this possibility. Notably, the results of the study fail to support the hypothesis that a psychosocial impact of allergies increases the risk of major depression.

While this study provides the first longitudinal analysis of a population sample, the study design was not capable of precisely timing the onset of depressive episodes nor of new onset allergies, therefore, temporal relationships were not necessarily definitively disentangled. In addition, the major depression measure in the NPHS, the CIDI-SF is a brief instrument that may not be as accurate as more detailed research diagnostic instruments [37,38]. Misclassification bias resulting from diagnostic inaccuracy may have caused a dilution in the observed strength of association, and this may have contributed to the lack of any apparent association between food allergies and major depression. The validity of the self-reported allergies is also questionable, but reproduction of a previously reported protective effect of smoking provides some evidence of methodological integrity. Since no direct assessment of allergy status was carried out, it is possible that health care seeking due to depression increased the probability of receiving a diagnosis of non-food allergies, rather than of developing allergies per se.

## Conclusion

These results indicate that people with major depression are at increased risk of developing non-food allergies. This may be due to an effect of major depression or to one or more shared risk factors.

## Competing interests

This analysis was based on data collected by Statistics Canada, but the results and interpretations do not reflect the opinions of Statistics Canada.

## Authors' contributions

SBP and ME jointly prepared the grant application and initiated the project. JVA and DHL contributed to the design of the study and analysis of data. All four authors contributed to the writing of the manuscript.
